# A multi-center, controlled, randomized, open-label clinical study of levofloxacin for preventing infection during the perioperative period of ultrasound-guided transrectal prostate biopsy

**DOI:** 10.1007/s10096-016-2742-5

**Published:** 2016-08-16

**Authors:** L.-D. Qiao, S. Chen, X.-F. Wang, W.-M. Yang, Y.-J. Niu, C.-Z. Kong, W. Tang, X.-F. Gao, B.-K. Shi, Y.-Q. Na, X.-D. Zhang, J.-Y. Wang, Y. Zhang, Z. Chen

**Affiliations:** 1 Department of Urology, Beijing Tongren Hospital, Capital Medical University, No. 1 Dongjiaominxiang, Dongcheng District, Beijing, 100730 China; 2 Department of Urology, People’s Hospital of Peking University, Beijing, China; 3 Department of Urology, Tongji Hospital, Wuhan, Hubei China; 4 Department of Urology, The Second Affiliated Hospital Tianjin Medical University, Tianjin, China; 5 Department of Urology, The First Affiliated Hospital of China Medical University, Shenyang, China; 6 Department of Urology, The First Affiliated Hospital of Chonqing Medical University, Chonqing, China; 7 Department of Urology, Changhai Hospital, Shanghai, China; 8 Department of Urology, Qilu Hospital of Shandong University, Shandong, China; 9 Department of Urology, Wu Jieping Urology Center of Peking University, Peking, China; 10 Department of Urology, Beijing Chao-Yang Hospital, Capital Medical University, Beijing, China; 11 Department of Urology, Beijing Hospital, Ministry of Health, Beijing, China; 12 Department of Urology, Beijing Tian Tan Hospital, Capital Medical University, Beijing, China; 13 Department of Medical Research Statistical Center, Fuwai Cardiovascular Disease Hospital, CAMS, Beijing, China

## Abstract

By comparing the safety and efficacy of 500 mg of oral levofloxacin for 3 days with those of intravenous antibiotics for 3 days in the prevention of infectious complications of ultrasound-guided transrectal prostate biopsy (TPB), we provided a safe and cost-effective infection preventive protocol for TPB in China. A total of 801 patients with indications for TPB in 12 centers were randomized into two groups from October 2011 to December 2015. Patients in the test group (*n* = 392) took 500 mg of oral levofloxacin for 3 days. Patients in the control group (*n* = 409) underwent intravenous antibiotics according to the traditional habits of the center for 3 days. All patients underwent ultrasound-guided TPB. Infectious complications were compared between the two groups. Different kinds of antibiotic were used in the control group. Comparing the two groups, the mean patient age was 70.6 ± 14.0 and 70.5 ± 14.0 years. The incidence of total infectious complications was 4.6 % (18/392) and 4.4 % (18/409) respectively, the incidence of asymptomatic bacteriuria was 3.1 % (12/392) and 2.7 % (11/409), the incidence of symptomatic urinary tract infection was 0.0 % and 0.2 % (1/409), the incidence of fever was 0.8 % (3/392) and 0.5 % (2/409), the incidence of bacteremia was 0.5 % (2/392) and 0.0 %, and the incidence of urosepsis was 0.3 % (1/392) and 1.0 % (4/409) respectively (all *P* > 0.05). The selection of antibacterial agents for TPB is in ca haotic condition in China. Oral levofloxacin at 500 mg once daily for 3 days is a safe, convenient, and cost-effective infection preventive protocol for TPB in China.

## Introduction

As the incidence of prostate cancer increases year by year in China, ultrasound-guided transrectal prostatic biopsy (TPB) has become an essential diagnosis method for prostate cancer. The use of prophylactic antibacterial agents plays an important role in reducing postoperative infective complications of TPB; however, there is no agreement on drug choice and course of treatment [1], and the choices of antibacterial agents, administration route, and course of treatment are all in disorder in China, which requires more multi-center studies to provide basis for clinical practice. To prove the efficacy and safety of levofloxacin 500 mg, po, qd, for 3 consecutive days as a prophylactic drug in the TPB, we conducted this study in the department of urology of many centers in China from October 2011 to December 2015. We already published the early result report of 296 cases in eight centers in China [2]; this report further expands to 820 cases in 12 centers based on the early study. This study has been reviewed and approved by the Ethics Committee of Beijing Tongren Hospital, Capital Medical University.

## Materials and method

### Trial design

This is a prospective, multi-center, randomized, control, open-label clinical study. From October 2011 to December 2015, patients suspected of prostate cancer requiring TPB were included in this study in the department of urology of 12 centers in China. The subject should meet one of the following criteria: positive result by digital rectal examination, PSA > 4 ng/ml, or radiographic examination as suspected prostate cancer. The subject signed a written informed consent. The main exclusion criteria included: patients with preoperative positive urine culture (colony count ≥10^5^ CFU/ml), preoperative pyuria (routine urine test > 5 WBCs/HPF), preoperative fever, disease causing low immunity or patients using immunosuppressors, coagulation disorders, severe cardiopulmonary insufficiency, abnormal liver function (ALT or AST > 2 × upper limits of normal, ULN), abnormal renal function (serum creatinine > 1.5 × ULN), patients allergic to test drugs, patients having taken antibacterial agents within 2 weeks before the inclusion, patients with preoperative indwelling urinary catheter, or any condition that the investigator considered to potentially increase the subject’s risk or interfere the test results.

The bowel preparation was performed by a uniform method: orally taking metronidazole 400 mg TID 2 days before biopsy, and discontinuing on the day of biopsy. In addition, coloclysis was performed with enema (110 ml glycerol enemas) before the biopsy. The methods were all ultrasound-guided TPBs with 10–13 punctures.

### Test drugs and dosage regimen

A total of 820 subjects were included in the study, and randomized into test group or control group. Patients in the test group orally took levofloxacin 500 mg [manufacturer: Daiichi Sankyo Pharmaceutical (Beijing) Co., Ltd; trade name: Cravit; Batch No.: 1106G18] on the day of biopsy (1–6 h before the biopsy) and the first and second day after the biopsy, while patients in the control group were given intravenous antibacterial agents according to the traditional habits of the center (excluding levofloxacin intravenous injection 500 mg, QD) on the day of biopsy and the first and second days after the biopsy.

### Study procedures

At 1–2 days before the biopsy, the patient’s demographic characteristics and laboratory test were recorded and then performed the bowel preparation. On the day of biopsy, the subjects were given test drug or control drug according to the results of randomized grouping. Blood culture was performed 2 h after the biopsy. Patients were followed up on days 1, 2, 3, and 7–10 after the biopsy. Blood routine, urine routine, and urine etiological examinations were performed on day 1. On day 7–10, the blood routine, urine routine, and routine biochemical tests were retested, and the blood or urine bacteriology was retested appropriately.

### Efficacy and safety evaluation

On day 7–10 after the biopsy, the investigator evaluated the following indexes:

Primary endpoints: (1) asymptomatic bacteriuria (ASB); positive urine culture on the morning of the second day after the biopsy, but the patient had no clinical symptoms, (2) symptomatic urinary tract infection (symptomatic UTI); patient with positive urine culture, pyuria, and symptoms of UTI, (3) fever; body temperature above 38 °C and excluding other infectious diseases, (4) urosepsis; presenting clinical symptoms of UTI accompanied with systematic inflammatory response syndrome, (5) bacteremia; blood culture detects pathogenic bacteria. Secondary endpoints: (1) use of antibacterial agents in control group, (2) cost of antibacterial agents and the drugs economic benefit cost ratio, (3) incidence of adverse drug reaction.

### Statistical method

The statistical analysis used SAS 9.2 to conduct the analysis; quantitative statistics were represented by mean ± SD, and conducted the *t*-test was conducted; the chi-square test was applied to enumeration data, with *P* ≤ 0.05 defined as significance.

#### Cost-effectiveness analysis and sensitivity analysis between the two groups

Cost-effectiveness ratio (C/E) represents the cost per unit effectiveness; a lower ratio means the cost of increasing by 1 unit of effectiveness is lower, and that the regimen is more significant in practice. The incremental cost-effectiveness ratio (∆C/∆E) represents the result of cost-effectiveness comparison between the two groups.

Sensitivity analysis was used to verify the impact of different hypotheses or estimates on analysis results. In this analysis, with other costs assumed to be constant, the drug price decreased 10 %.

## Results

A total of 19 of the 820 patients were excluded from the analysis set due to noncompliance with the main inclusion criteria. Finally, there were 801 evaluable patients, 392 patients in the test group and 409 patients in the control group. See Table 1 for the patients’ basic clinical data for the two groups. There was no statistically significant difference between the two groups for all parameters. (Table 1)

**Table 1:** Patients’ demographic and baseline characteristics data

Index	Test group	Control group	*P* value
	(*n* = 392)	(*n* = 409)	
Age (year)	70.6 ± 14.0	70.5 ± 14.8	0.512
Height (cm)	170.2 ± 5.9	170.2 ± 5.8	0.832
Weight (kg)	71.0 ± 10.0	71.0 ± 5.8	0.926
Course of Disease (month)	0.6 ± 2.1	0.5 ± 2.2	0.421
Positive DRE	141 (36.0 %)	141 (34.5 %)	0.658
PSA (ng/ml)	50.1 ± 367.4	49.9 ± 368.3	0.446

DRE: digital rectal examination; PSA: prostate specific antigen

Notes: no difference between two groups for other demographic indicators (body temperature, heart rate, respiratory rate, blood pressure, etc.)

1. Table 2 shows the use of antibiotics in the control group.

**Table 2:** Drugs list for the control group

Type of antibiotics	Representative drug	Injection/PO	*N* (%)
Second-generation cephalosporins	Cefotiam	Injection	135 (33.0 %)
Quinolones	Ciprofloxacin	Injection	118 (28.9 %)
Third-generation cephalosporins	Ceftriaxone	Injection	19 (4.6 %)
Monocyclic β-lactam	Aztreonam	Injection	28 (6.8 %)
Compound preparations of penicillins	Pipercillin /tazobactam	Injection	41 (10.0 %)
Aminoglycosides	Streptomycin	Injection	7 (1.7 %)
Cephamycins	Cefmetazole	Injection	61 (14.9 %)
Total			409
2. The overall incidence of infective complications was 4.5 % (36/801), including ASB 2.9 % (23/801), symptomatic UTI 0.1 % (1/801), fever 0.6 % (5/801), bacteremia 0.3 % (2/801) and urosepsis 0.6 % (5/801). Comparison of the infective complications for the two groups is shown in Table 3. There was no statistically significant difference for all infective complications (for all results, *P* > 0.05). The results of stratified comparison of infectious complications among the top three drugs (cefotiam, ciprofloxacin and cefmetazole) in the control group and the test group are shown in Table 4. The three drugs were respectively compared with levofloxacin (500 mg po qd), and there was no statistical difference in the incidence of infectious complications (*P* > 0.05).

**Table 3:** Comparison of incidence of postoperative infective complications between two groups

	Infective leftcomplications (%)	ASB (%)	Symptomatic UTI (%)	Fever (%)	Bacteremia (%)	Urosepsis (%)
Test group (*n* = 392)	18 (4.6 %)	12 (3.1 %)	0	3 (0.8 %)	2 (0.5 %)	1 (0.3 %)
Control group (*n* = 409)	18 (4.4 %)	11 (2.7 %)	1 (0.2 %)	2 (0.5 %)	0	4 (1.0 %)
*P value*	0.8963	0.7528	0.3273	0.6197	0.1481	0.1941

ASB: asymptomatic bacteriuria; UTI: urinary tract infection

**Table 4:** Comparison of incidence of postoperative infective complications between levofloxacin and other three antibiotics

	Infective complications (%)	ASB (%)	Symptomatic UTI (%)	Fever (%)	Bacteremia (%)	Urosepsis (%)
Levofloxacin (*n* = 392)	18 (4.6 %)	12 (3.1 %)	0	3 (0.8 %)	2 (0.5 %)	1 (0.3 %)
Cefotiam (*n* = 98)	4 (4.1 %)	3 (3.1 %)	0	1 (1 %)	0	0
*P* value*	0.8273	1		0.8018	0.4786	0.6142
Ciprofloxacin (*n* = 86)	4 (4.7 %)	3 (3.5 %)	0	0	0	1 (1 %)
*P* value*	0.981	0.837		0.4157	0.5068	0.2376
Cefmetazole (*n* = 46)	1 (2.1 %)	1 (2.1 %)	0	0	0	0
*P* value*	0.4463	0.7369		0.5516	0.6273	0.7316

*compare with levofloxacin

ASB: asymptomatic bacteriuria; UTI: urinary tract infection
3. The cost of antibacterial agents of a total of 707 patients was summarized: 345 cases in the test group and 362 cases in the control group. The mean cost in the test and control groups was 43.0 ± 3.8 and 402.7 ± 279.2 CNY (1 CNY = 0.1611 USD) respectively (*P* < 0.001). Table 5 showed the results of mean costs of the two groups and the cost-effectiveness analysis and sensitivity analysis between the two groups.

**Table 5:** Cost effectiveness analysis

		Cost (yuan, C)	Effectiveness (%, E)	C/E	△C/△E
Test group		43.05	95.4	45.1	179825
Control group		402.7	95.6	421.2	
Test group	Sensitivity analysis	38.745	95.4	40.6	161843
Control group	Sensitivity analysis	362.43	95.6	379.1	

Effectiveness = 1 – percentage of infective complications, i.e., the percentage of no infective complications

C/E: cost effectiveness ratio, CER

△C/△E: incremental cost effectiveness ratio, ICER

Sensitivity analysis: cost decreased 10 %
4. Safety analysis

The safety analysis set included 820 patients: 410 patients in the test group and 410 patients in the control group. No drug-related adverse events occurred in either group.

## Discussion

While using prophylactic antibacterial agents, the incidence of postoperative infective complications of TPB still exists [3], including the incidence of symptomatic UTI 2–6 % [4] and urosepsis 0.1–2.2 % [5]. Our results showed that for patients with preoperative clean urine, using prophylactic antibacterial agents and a uniform bowel preparation method, the incidence of postoperative infective complications was almost similar to that from international reports.

This study also found the current chaotic condition in China when urologists selected antibacterial agents for TPB. Based on our study results, 2nd generation cephalosporin (cefotiam, cefuroxime or the others) had the highest proportion selected by urologists, followed by fluoroquinolones. This is possibly because Chinese drug resistance data showed that the fluoroquinolone resistance rate of *Escherichia coli* in male urine sample had exceeded 50 % [6], thus many urologists lacked confidence in fluoroquinolones. Some urologists selected compound preparations of penicillins and cephamycins (cefmetazole or cefoxitin), possibly because of the excessively high isolation rate of the strain producing extended spectrum β lactamases in China [6]. But it should be noted that all resistance data in China were mainly from the bacteria of hospital-acquired UTI, not the resistance of community-acquired infectious bacteria, and in addition, the prophylactic medication was different from treatment medication, so these high resistance data didn’t completely apply to the use of prophylactic antibacterial agents. Chiang et al. also proved that although the fluoroquinolone resistance rate of *Escherichia coli* increased, the clinical efficacy showed that fluoroquinolones were still the main drugs to prevent postoperative infective complications of TPB. The high resistance data indicate that once infective complications occurred, fluoroquinolones would not be appropriate to be used in further treatment [7]. Levofloxacin (500 mg, po) shows favourable pharmacokinetics, excellent penetration into the prostate, good bioavailability and equivalent oral and parenteral pharmacokinetics which completely complied with the requirements of the TPB procedure [8]. Our results also showed that compared with clinically common prophylactic regimens of various intravenous antibacterial agents, levofloxacin 500 mg, po, qd, for 3 consecutive days showed the same effect in preventing infective complications, with lower cost and economic advantage. The disadvantage of this study was lack of uniform selection of the antibacterial agent for the control group. In the future, we plan to further conduct head-to-head controlled studies of levofloxacin in comparison with different drugs, and study different courses of treatment with levofloxacin.

In conclusion, from the results we can see that the selection of antibacterial agent for TPB is in a chaotic condition in China. We prove that levofloxacin 500 mg po qd for 3 days is a safe, effective, convenient, and proper prophylactic protocol with optimal economic benefit cost ratio in the prevention of infective complications of TPB in China. To our knowledge, this is the first multi-center study in China in this area.
